# Sun-Exposure-Related Healthcare Use: Analysis of Pharmacy Sales, SOS Médecins Records, and Emergency Department Visits: PRISME Study, Occitanie, Southern France, 2019–2022

**DOI:** 10.3390/ijerph23040476

**Published:** 2026-04-09

**Authors:** Leslie Simac, Olivier Catelinois, Yasmine Yahiaoui, Franck Golliot, Damien Mouly

**Affiliations:** 1 Santé Publique France, Regions Division, 34000 Montpellier, France; olivier.catelinois@santepubliquefrance.fr (O.C.); yasmine.yahiaoui@bsci.com (Y.Y.); franck.golliot@santepubliquefrance.fr (F.G.); 2 Santé Publique France, Regions Division, 31000 Toulouse, France; damien.mouly@santepubliquefrance.fr

**Keywords:** sunburn, sun-overexposure, solar UV, health impact, health care use, health care utilization, pharmacy, medicine, emergency department

## Abstract

**Highlights:**

**Public health relevance—How does this work relate to a public health issue?**

- Skin cancers are increasing, with a significant proportion linked to sun exposure during childhood.
- Sun-exposure-related cares can be avoided through sun-protective behaviors.

**Public health significance—Why is this work of significance to public health?**

- This study shows that sun-exposure-related care increases from from early spring.
- The multi-source approach highlights significant differences in healthcare utilization.

**Public health implications—What are the key implications or messages for practitioners, policy makers and/or researchers in public health?**

- This study demonstrates the importance of non-reimbursed medications in measuring the impact of symptoms without severity criteria.
- Pharmacy sales can be useful for monitoring effectiveness of prevention measures in a population.

**Abstract:**

UV radiation causes health effects and repeated excessive sun exposure during childhood increases the risk of skin cancer in adulthood. The French region of Occitanie combines conditions conducive to sun exposure with a wide range of healthcare services. The study aims to describe temporal variations related to sun overexposure and patient characteristics, and evaluate the relevance of each data source. We conducted a retrospective analysis (2019–2022) on pharmacy sales, emergency care provided by SOS Médecins (SOSM), and emergency departments (EDs). More than 220,000 customers purchased products associated with sun overexposure, while 71 SOSM procedures and 417 ED visits were recorded. The activity is clearly seasonal, but remains five to ten times higher for pharmacies than for other sources. About 80% of ED patients were under 40 years of age, while 50% lived within 20 km of the consultation location. The impacts on healthcare systems vary, and each provides complementary insights into care related to sun overexposure. Increases in pharmacy sales are observed as early as spring, underscoring the need to strengthen prevention messaging from the start of the season. The study confirms the value of pharmacy sales data for assessing the impact of sun exposure, but ED or SOSM data enable real-time monitoring and patient characterization.

## 1. Introduction

Ultraviolet (UV) radiation—an invisible component of the electromagnetic spectrum emitted by the sun—does not produce heat. Although most people (9 out of 10) recognize sun exposure as a probable cause of skin cancer [1], tanning remains a perceived sign of beauty [2]. The World Health Organization (WHO) highlights the beneficial effects of moderate UV exposure, particularly their essential role in vitamin D synthesis, but emphasizes that excessive exposure can have acute or chronic health effects, including dermatological, ocular, or immune system disorders [3]. The onset of skin cancer in adulthood may be linked to excessive exposure to the sun during childhood or adolescence. Sun exposure is therefore a major public health issue, and preventive measures must be applied from childhood and throughout life.

Solar erythema or sunburn is skin damage directly linked to exposure to solar UV radiation and may involve one or more areas of the body, with or without blistering, depending on the severity. The onset is rapid: dermatological effects occur within 12 to 24 h of excessive sun exposure [4,5]. To date, few scientific studies have examined healthcare utilization related to the short-term effects of solar UV exposure. The international literature reports two study methodologies concerning acute dermatological effects associated with the sun: studies based on consultations or hospitalizations in healthcare facilities [6,7,8,9,10,11,12], and studies based on field surveys [4,13,14,15,16]. However, the latter requires significant human and financial resources and is further limited by the fact that studies are conducted in restricted areas and over short time periods, making it difficult to account for weather-related uncertainties. Studies based on passive data collection (medical administrative databases) focus mainly on emergency department (ED) visits [6,7,8,10,11] and, less frequently, on hospitalizations [7,12]. This observation confirms the relevance of studying the impact of sun-related dermatological effects on healthcare utilization in France.

The French region of Occitanie, bordered by the Mediterranean Sea, has a 200 km coastline (geographic coordinates for Le Grau-du-Roi, the northernmost town, are 43°32′17.0″ N, 4°08′14.2″ E; for Cerbère, the southernmost town, the coordinates are 42°26′39″ N, 3°09′56″ E). The climate is highly favorable for sun exposure, with a large number of sunny days in its coastal departments: from 196 to 238 days in 2021 [17]. In addition, the region attracts many tourists during the summer, with over 222 million overnight stays recorded in 2023 [18].

The Occitanie region presents a combination of factors that are conducive to studying the impact of sun exposure on healthcare systems, abundant sunshine, substantial summer tourism, and year-round water and outdoor activities, all of which are strongly associated with sunburn as reported in the sun survey conducted by Cardinez et al. [4].

The work presented here is part of the PRISME (Prevention and Impact of Sun Exposure on the Mediterranean Coast) project, which aims, on the one hand, to guide actions to reduce the health impact of sun exposure by adapting prevention campaigns (prevention component [16]), and, on the other hand, to better describe the health impact of short-term sun exposure (impact component). Following a pilot study to develop an impact indicator based on pharmacy product sales [19], the present study estimates the impact of excessive sun exposure on different types of healthcare use: products purchased in pharmacies to alleviate the effects of sunburn, and unscheduled care in general practices or hospitals. The objectives of this multi-source approach are to (i) describe temporal variations in healthcare utilization related to sun exposure, both in terms of frequency and patient demographics, and (ii) assess the relevance, advantages, and limitations of each data source for assessing the impact of sun exposure.

## 2. Materials and Methods

**Figure 1:**
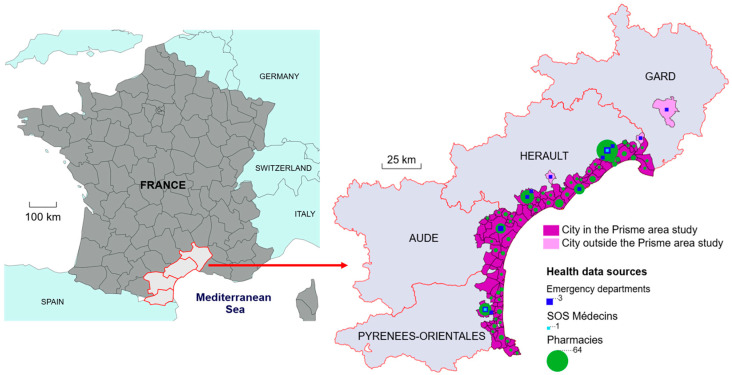
Distribution of pharmacies, SOS Médecins associations, and emergency departments by municipality of the PRISME project study area in France.

The study area covers the entire Mediterranean coastline of the French region of Occitanie and includes 94 municipalities (shown in purple in Figure 1).

The study population includes all individuals who received healthcare directly in or near the study area, taking into account the healthcare utilization patterns of residents in the study area (municipalities marked in purple or pink in Figure 1) during the target period.

The study period is from 2019 to 2022.

The study focuses on data from pharmacies as well as unscheduled urgent care in general practices and EDs, motivated by a study conducted in the United States among beachgoers with sunburns, which found that 34.3% of individuals used over-the-counter (OTC) medications, while only 0.3% had a prescription, 0.3% consulted a healthcare professional, and 0.04% went to an ED [13].

### 2.1. Sale of Products in Pharmacies

In France, OTC medicines are only available in pharmacies. These form a dense territorial network since, according to the National Council of the Order of Pharmacists (NCOP), there is one pharmacist for every 2600 inhabitants. With 385 pharmacies open during the study period, the study area has approximately one pharmacy for every 2800 inhabitants. The 11 pharmacy management software (PMS) publishers recommended by the NCOP and approved by the Digital Health Agency [20] were invited to participate in our study. Specifications were provided to enable framing and standardization of the data. Two publishers signed contracts with Santé publique France, the French national public health agency, covering 69% of the targeted pharmacies, or 265 pharmacies. Data were provided for 244 of them. As the completeness of historical data varied between pharmacies, a sample was created including only those that had transmitted at least 80% of weekdays for each year, excluding Sundays and public holidays. The final dataset included 115 pharmacies for 2019 (30% of the 385 pharmacies in the study area), 203 (53%) for 2020, 218 (57%) for 2021, and 232 (60%) for 2022. All 232 pharmacies are mapped at the municipal level in Figure 1.

Preliminary work was necessary to define the indicators to be collected in pharmacies: the list of products to be targeted was based on a pilot study conducted for our project [20], which categorized products into two lists according to their positive predictive value (PPV). The most specific indicator, i.e., the one with a PPV greater than or equal to 70%, was selected, corresponding to products for which purchases were reported as linked to sunburn in 70% or more of the questionnaires from the pilot. This list includes around 50 products for treating or alleviating the effects of sun overexposure, including medicines subject to marketing authorization (MA) in France and medical devices (OTC products not subject to the MA process in France). In the extraction requested from publishers, the number of customer receipts reporting the sale of at least one of these products was requested for each day, along with the total number of customers for the day, regardless of whether the purchase was prescription or OTC. Thus, the proportion of suncare activity represented the number of customer receipts including a suncare product from the list, relative to the total number of receipts, expressed per 1000 customer receipts (=PA_pharma_). This indicator is available in aggregate form by day and by pharmacy and does not contain any individual patient information.

### 2.2. Emergency Care in Town or Hospitals

The SurSaUD^®^ (Health Surveillance of Emergencies and Deaths) system from Santé publique France provides an overview of unscheduled and emergency care in general medicine, as well as hospital ED activity. This information system integrates four data sources, including the Oscour^®^ network (hospital ED) and SOS Médecins (associations of physicians responding to general medical emergencies [21]). It enables Santé publique France to perform real-time syndromic surveillance with multiple objectives: identifying unexpected events, monitoring trends such as seasonal epidemics and assessing the impact of environmental or infectious phenomena. These data can also offer insights into the short-term dermatological effects of sun exposure.

SOS Médecins (SOSM) associations provide emergency medical services by visiting people at home or seeing patients in dedicated centers. Two such facilities are located directly in the study area (Figure 1), each with consultation centers where patients can schedule appointments. The data transmission rate for these associations was approximately 100% per year, given that they are open every day. SOSM data contain both administrative data (date and time of the call, age and sex of the patient, residence location) and medical data (main diagnosis and one or more associated diagnoses).

In the thesaurus used by the two SOSM associations in question, a single code corresponds to sunburn. The SOSM Montpellier association was excluded from the analyses as it did not use the code dedicated to sunburn. Two indicators were collected for the SOSM Perpignan association: the daily number of sunburn cases and the total number of medical procedures performed during the day. This allowed the calculation of the proportion of medical procedures for sunburn among all procedures, regardless of the cause, expressed per 1000 medical procedures to enable comparisons between sources (=PA_SOS_). This indicator is available daily.

Most hospital EDs are members of the Oscour^®^ network [21] and transmit data daily in the form of emergency visit summaries (EVSs). These summaries contain both administrative data (date and time of visit, patient’s date of birth and gender, residence location) and medical data (main diagnosis and one or more associated diagnoses, severity and patient discharge details). The Occitanie region has 68 EDs, including both public and private healthcare facilities. EDs were selected based on the assumption that the healthcare-seeking habits of local residents are similar to those of tourists in the area. A method for identifying the habits of residents was described by Pouey et al. [22] using postal codes in EVSs (one code can correspond to one or more municipalities). This enabled the selection of 16 EDs in or near the study area (Figure 1), including two university hospitals (one located outside the study area), four other public facilities, and ten private clinics. These facilities are open every day of the year, generally 24 h a day, and data were available in the Oscour^®^ database for 94 to 100% of days, depending on the facility and the year in question.

For emergency medical data, a Delphi survey [23] was used to validate an indicator for monitoring the effects of sun exposure by healthcare professionals. The ICD-10 (10th revision of the International Classification of Diseases) codes retained were: L55 (sunburn), L56 (other acute changes in the skin due to ultraviolet rays) and X32 (exposure to sunlight), the latter indicating the circumstance rather than the effect. Although most previous studies [6,8,10,11] relied solely on the main diagnosis and focused on ICD-9 equivalents of L55 codes, we broadened the approach by querying codes L55, L56, or X32 in both the main and associated diagnoses. The two indicators extracted were the daily number of visits related to sun and the total number of visits per day, from which we calculated the proportion of activity attributable to sun exposure per 1000 EVSs, regardless of the cause, for comparison with other sources (=PA_ED_). This indicator is available by hospital on a daily basis. EVSs can be used to describe the characteristics of patients (age, gender, and residence location). Ages are analyzed by age group: 0 to 9 years for children, 10 to 19 years for adolescents (referring to the WHO definition of adolescents [24]), and 20 to 39 years old, 40 to 59 years old, and 60 years old or older for adults. To analyze emergency data based on patient origin, the distance between residence and consultation location was calculated using the geographical coordinates of the centroid of the patient’s municipality of residence and those of the municipality where the ED is located, converted into kilometers. Distances were analyzed according to the following categories: 0 to 19 km, 20 to 49 km, 50 km and above, and “Outside the area,” i.e., postal codes beginning with 99, generally indicating a foreign country or an unreported/incorrectly reported code.

### 2.3. Statistical Analyses

Weather conditions can vary substantially every year: early onset of sunny weather, summer storms, overcast conditions, sea breezes, and wind patterns may differ between the southern and northern zones. To account for these potential climatic variations, daily indicators were analyzed separately for each year from 2019 to 2022. In particular, we compared data for the meteorological summer (1 June to 31 August [25]) with data from the other months of the same year (1 January to 31 May and 1 September to 31 December).

Boxplots were used to analyze the monthly distribution of indicators. They allow visualization of the first quartile, median, mean, third quartile, minimum and maximum for each month, as well as outliers (values beyond Q1 − 1.5 × IQR or Q3 + 1.5IQR, where IQR is the interquartile range between Q1 and Q3).

## 3. Results

Across the four-year period, the pharmacies included in the study generated approximately 54 million customer receipts (Table 1), the SOSM association in Perpignan recorded fewer than 200,000 emergency medical procedures, and the 16 targeted EDs documented nearly 3 million visits.

Considering only remedies that may be related to sun exposure, the volumes are significant: more than 220,000 pharmacy customers purchased at least one of the suncare products on the list from 2019 to 2022 in the targeted pharmacies (Table 2). Over the same period, SOSM Perpignan recorded 71 medical procedures for sunburn and the 16 selected EDs documented 417 EVSs. The maximum daily counts were 1435 customers purchasing at least one product from the list (June 2021), three procedures at SOSM and six EVSs.

The proportion of activity represented by these incidents varies substantially depending on the year, data source, and season (Table 2). Over the four summers studied, approximately 11 out of every 1000 pharmacy sales were for a suncare product (ranging from 10 to 12 depending on the year; PA_pharma_), while only one medical procedure in 1000 involved sunburn for SOSM (ranging from 0 to 2 per summer; PA_SOSM_), and less than one ED visit in 1000 involved sunburn (PA_ED_). The use of pharmaceutical products is relatively common, reflecting the generally low severity of sun exposure short term. Confirming this low severity, it should be noted that only 2% of patients who visited the ED for a sun-related condition were hospitalized, whereas, for all medical causes, approximately 15% of patients are hospitalized after visiting an ED.

**Figure 2:**
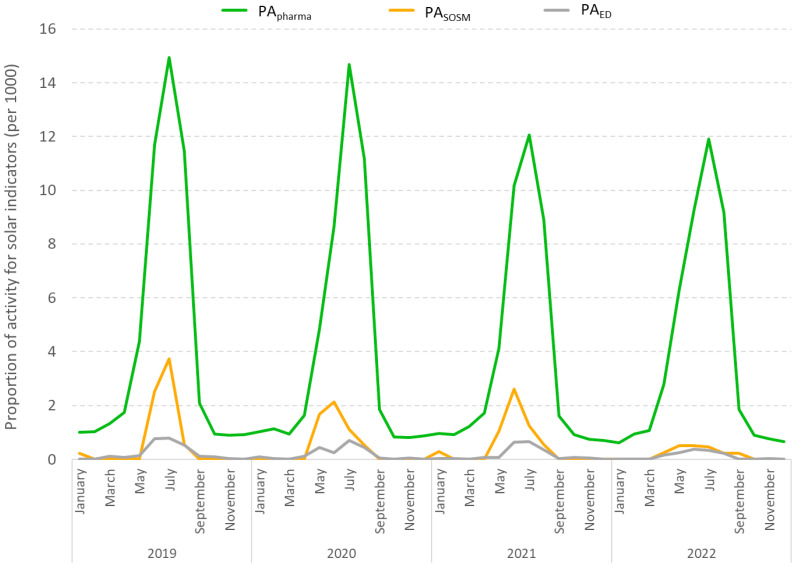
Proportion of monthly activity related to specific sun exposure indicators for each data source (PA_pharma_, PA_SOSM_, PA_ED_) relative to total activity (per 1000 pharmacy customer receipts, per 1000 SOS Médecins procedures in Perpignan, and per 1000 emergency department visits), January 2019 to December 2022.

For all three data sources, the proportion of monthly activity varies considerably throughout the year, with the lowest values observed in winter and the highest in summer months (Figure 2). For example, the proportion of pharmacy customer receipts that include at least one suncare product ranges from 12 to 15 per 1000 customers over the four summers analyzed, while it is ten times lower outside summer months. For the other two sources, during summer, 0.5 to 3.7 per 1000 SOSM procedures involve sunburn, and just 0.2 and 0.8 per 1000 EVSs are related to sun exposure. Overall, the proportion of care related to sun exposure during summer is five to ten times higher in pharmacies than in SOSM or EDs.

**Figure 3:**
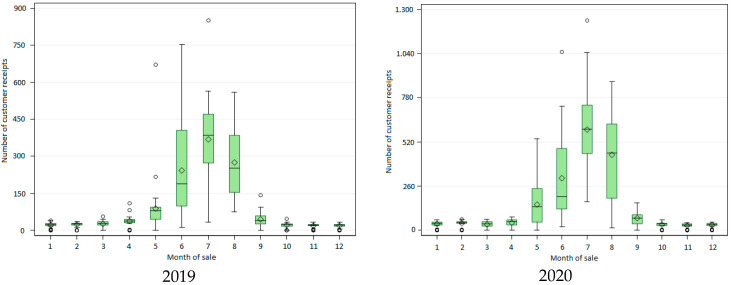

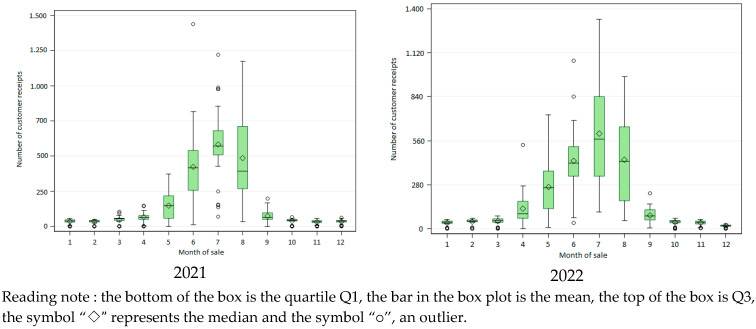
Monthly distribution of daily pharmacy customer receipts including suncare product sales, 2019–2022.

Sales growth follows different patterns depending on the year: from May to July for 2019, 2020 and 2021, but starting as early as April in 2022. Figure 3 also shows that peak daily sales are not always observed in the same month: July in 2019, 2020, and 2022, but June in 2021.

The SOSM data were limited (N = 71) and focused on a small part of the study area, making them insufficiently representative for analysis by patient gender, age, or residence location.

The male-to-female ratio for ED visits due to sun-related issues varied annually, from 0.8 (2019) to 1.2 (2020). Across the four-year period, the overall ratio was 0.95, compared to 1.03 for all EVSs.

Each year, the youngest patient admitted to the ED for sunburn was under 1 year old, while the oldest ranged from 72 to 85 years old, depending on the year. Median age varied from 19 to 22 years old. Over the four years analyzed, approximately 50% of patients were under 20 years old: children (0–9 years old) accounted for 19% of visits (79 EVSs), and adolescents (10 to 19 years old) also accounted for 19% (124 EVSs) (Table 3).

Patients residing less than 20 km from the consultation site accounted for more than 50% of EVSs in 2019, 2020 and 2022 (Table 3). In contrast, in 2021, the majority of patients (54% of EVSs) resided more than 50 km from the consultation site.

**Figure 4:**
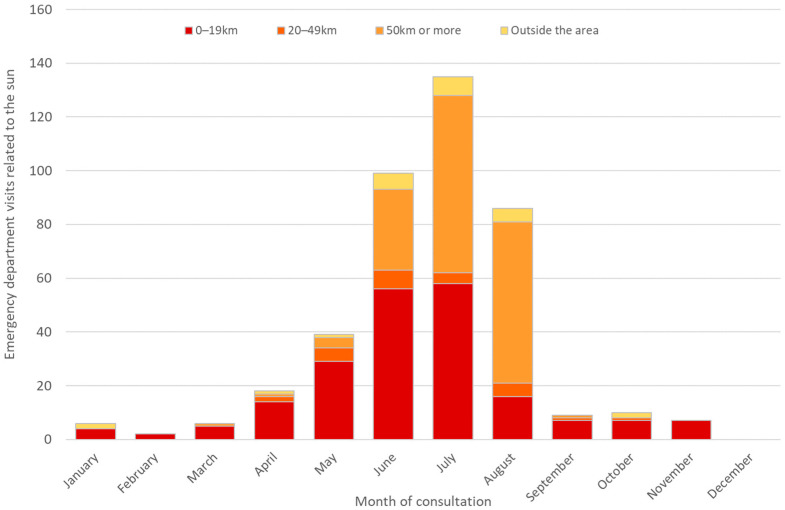
Origin of emergency department (ED) visits for sun-related conditions by distance from residence to ED location, by month of consultation. Data are cumulative for 2019–2022 across the 16 EDs in the study area.

Patients consulting for sunburn mainly come from towns located less than 20 km from the consultation location in April, May, and June. During the summer period, however, the proportion of patients residing more than 50 km away or outside the area increases significantly, reaching 76% ED visits for sunburn in August, compared with 54% in July and 36% in June (Figure 4).

To complete the analysis, an examination of the associated diagnoses recorded in EVSs showed that the coded symptoms are consistent with sun overexposure (headaches, hyperthermia, and dizziness in particular), or with burns (head, lower limbs) used in association with L55/sunburn. ICD-10 codes T20 to T25, representing burns on the external surface of the body, and T29 to T31, representing burns on multiple or unspecified body parts (excluding subcodes for corrosion), are widely used by certain EDs. These codes do not specify the agent responsible for the burn; however, code T31, which classifies burns according to the extent of the body surface area affected (10%, 20%, 30%…), is very often reported.

## 4. Discussion

The impact component of the PRISME project provides new insights into healthcare consumption related to sun exposure over a four-year period, using data on unscheduled visits to general practices and hospitals, as well as sales of non-reimbursed medications in pharmacies. These healthcare systems are affected differently: while all data generally show a seasonal peak during summer, pharmacy sales exhibit an earlier increase. ED data also allowed identification of patient profiles: the vast majority are under 40 years of age and typically reside near the consultation location, except in July and August, when many patients are tourists.

The results of this study highlight the impact of sun exposure on local healthcare systems, even though these effects are largely preventable through appropriate behaviors [16]. The burden varies greatly across sectors, with pharmacies experiencing ten times more activity related to sun exposure during the summer than EDs or the SOSM association. This finding is consistent with the study by DeFlorio-Baker et al. [13], which reported that individuals prefer to buy OTC products to manage sun overexposure. Our results therefore reinforce the central role of pharmacists in the management of sunburn. Pharmacy suncare product sales also show that seasonal increases begin before the summer period, typically in May or even April depending on the year. This earlier rise compared with other healthcare sources likely reflects the generally low severity of sun exposure effects, as evidenced by the very low proportion of hospitalizations among ED visits.

The DeFlorio-Baker study [13] shows that the likelihood of experiencing sunburn does not differ according to the distance from the beach. In our study, although the exact exposure sites are unknown, the distances between patients’ residences and consultation sites indicate that, in Occitanie, individuals living less than 20 km from an emergency facility account for a significant proportion of ED visits for sunburn from April to July. This is noteworthy given that, according to the 2021 Santé publique France barometer [26], the population of metropolitan France considers itself well informed about the health effects of sun exposure, with even higher levels of perceived knowledge among populations living near the coast. Occitanie is one of the fastest-growing French regions and has a particularly attractive coastline [27]. The continual arrival of new residents every year means that the “naive” population, or those less familiar with the local climatic conditions and the harmful effects of sun exposure, is regularly renewed in a region with many sunny days, where exposure is not limited to the summer holiday period. Our analyses also reveal the high proportion of tourists visiting EDs in July and August, when tourist numbers peak and solar UV radiation is high or very high. Furthermore, the COVID-19 pandemic seems to have had a limited impact on tourism along the Mediterranean coastline: although international tourism declined, this was partially offset by visitors from other regions of France.

Patients consulting EDs for sunburn are predominantly young, with nearly half of them under the age of 20, despite WHO warnings that excessive sun exposure in childhood and adolescence can lead to cancer in adulthood [3]. Our findings are consistent with the work carried out as part of the prevention component of the PRISME project by Durand et al. [14], which showed that tourists on the Occitanie coast who intentionally expose themselves to the sun for tanning and who report the lowest levels of protection are most often aged between 15 and 24. Similarly, the study by Tripathi et al. [8] found that approximately 80% of ED patients presenting with sunburn were under 40 years of age. This is in line with our data, in which the proportion ranges from 80% to 90% depending on the year.

The data sources used have many advantages but also present certain limitations. France has a dense network of pharmacies, making it possible to identify many treatments related to sun exposure. Frequently located near places of interest, such as beaches, where UV exposure is high, they provide access to advice, medical devices, and medications without requiring a physician’s consultation, especially since pharmacies in the study area extend their opening hours and days during the summer (opening on Sundays and public holidays). Because data were collected directly from PMS publishers, most pharmacies were able to participate in the study, resulting in robust coverage. The pharmacy sales data available for four consecutive years clearly illustrate seasonal variation: daily volumes are very low in the winter and rise sharply in the summer. However, differences were still observed between the same months across years, with a significant increase from April 2022 onwards, whereas in the other three years, the increase occurred in May. This difference could probably be explained by milder weather in 2022, conducive to earlier sun exposure. This hypothesis would need to be verified by comparing the findings with local weather data and this lack of comparison may be considered a limitation of our study, in which the observed seasonality is regarded as an indirect indicator of weather conditions.

The list of medicines and medical devices compiled by Riondel et al. [19] is a closed list. Because the range of suncare products is wide and constantly evolving, not all products can be taken into account. While medicines in France are sold exclusively in pharmacies, medical devices can also be purchased in drugstores, for which no data were collected as they do not use compatible software. Moreover, these types of data do not provide individual information on buyers. As a result, studying the profile of these individuals would require repeated surveys over several years, allowing comparison with the characteristics of patients seen in EDs.

Our study used data from SOSM associations, routinely available in the SurSaUD^®^ system of Santé publique France [21]. Their territorial coverage is limited, with 64 associations in France. Each association typically serves one or more municipalities within a large urban area for home visits, although patients are free to visit the medical center for a consultation following a call. Of the two associations initially targeted in the study area, one did not use the code for sunburn at all. Consequently, the data are not representative of the study area, and the volume of medical procedures is very low compared to other sources. Had the data been more representative, these medical procedures could have provided valuable information on patient characteristics (age, gender, and origin).

The distribution of EDs in the study area is heterogeneous, with most concentrated around large cities. Unlike other studies based on hospital data or a sample of establishments [6,7,8,10,28], the data extracted here can be considered nearly exhaustive, as it includes all visits to the EDs selected according to the habits of residents in the PRISME study area.

Finally, the analysis of diagnoses recorded in EVSs showed that emergency physicians may use other ICD-10 codes for burns beyond the specific codes targeted, which indicate one or more body parts but do not specify the causative agent. A study such as that by Xia et al. [8], which includes a review of patient files, could help estimate the PPV of each of these codes by distinguishing burns related to sun exposure from those caused by other agents, such as fire, a frequent summer hazard when lighting barbecues. Incorporating codes with a high PPV would help refine the query performed in our study.

There are two major differences between the three sources and the types of care concerned. First, ED and SOSM data are based on medical diagnoses, whereas pharmacy data reflect product purchases and therefore also represent self-medication in response to symptoms related to sun exposure. Second, although data from the SurSaUD^®^ system are available daily and can be used throughout France for real-time monitoring, the volume of cases is too low to allow reliable daily analyses. Pharmacy data, meanwhile, were collected from PMS publishers, providing robust characterization of seasonal impact but not supporting real-time monitoring. Finally, the timing of care or product use must be taken into account when interpreting the results. ED or SOSM visits generally occur close to the time of exposure, whereas pharmacy purchases may be preventive, occurring prior to the onset of health effects. Similarly, vacationers may bring this type of product with them in their first-aid kit, reducing local purchases and potentially leading to an underestimation of usage.

## 5. Conclusions an Outlook

The impact component of the PRISME project described the temporal evolution of healthcare utilization related to sun exposure along the Occitan coastline, including pharmacy sales, SOSM medical procedures, and ED visits. The study shows that healthcare utilization increases in the spring, peaks in the summer, and decreases in the fall, with the proportions varying greatly depending on the data source. Most patients seen in EDs were under 40 years of age, and half lived within 20 km from the consultation site, a proportion that decreased in the summer in favor of tourists, with these visitors also experiencing high solar UV exposure.

ED data, while less abundant than pharmacy sales data, are available in real time through automated systems and can be used to describe patient demographics and their geographic origin. However, the low volume of data does not allow for a very accurate assessment of trends, particularly when it comes to determining the early onset of sun-related health effects. In contrast, pharmacy sales data, which are much more frequent, provide a more accurate estimate of the temporal trend and short-term health impact of sun exposure. However, the way in which this data are collected (requiring a request to a PMS and associated costs) makes it difficult to use for real-time monitoring of sun-related health effects.

PRISME also highlights the value of data on sales of non-reimbursed medicines in pharmacies to monitoring the effects of solar UV radiation. Although such data are not used in routine monitoring in France, their importance is shown here for symptoms without severity criteria and that, in most cases, do not require medical consultation.

The National Cancer Institute launches its annual sun protection information and awareness campaign every year in May. Our findings highlight the need to disseminate prevention messages even earlier, to promote appropriate sun-protective behaviors and reduce the risk of acute effects of overexposure to solar UV rays, such as sunburn.

## Figures and Tables

**Table 1:** Annual counts of pharmacy customer receipts, SOS Médecins Perpignan medical procedures, and emergency department (ED) visits from the 16 EDs in the study area, with combined totals for the four-year study period (2019–2022).

Year	Pharmacy Customer Receipts	SOS Médecins Procedures	Emergency Department Visits
2019	7,894,884	43,775	713,652
2020	13,141,942	40,262	596,641
2021	15,702,675	44,128	690,629
2022	16,657,122	52,491	737,596
2019–2022	53,396,623	180,656	2,738,518

**Table 2:** Volume of data on sun-exposure-related healthcare use and its proportion of overall activity during summer and non-summer periods, from 2019 to 2022 and over the four-year period, in pharmacies, SOS Médecins Perpignan and emergency departments.

	Pharmacies		SOS Médecins		Emergency Departments	
Year	Customer Receipts with Sun Care Products	PA_pharma_	Procedures for Sunburn Erythema	PA_SOSM_	Emergency Visits Related to the Sun	PA_ED_
Outside of summer 2019 Summer 2019	9089	1.6	1	0.0	26	0.1
	27,309	12.8	23	2.2	110	0.6
Outside of summer 2020 Summer 2020	14,406	1.5	5	0.2	29	0.1
	41,396	11.7	12	1.2	68	0.4
Outside of summer 2021 Summer 2021	15,660	1.4	5	0.2	16	0.0
	45,868	10.3	16	1.4	89	0.4
Outside of summer 2022 Summer 2022	21,326	1.7	4	0.1	26	0.0
	45,301	10.2	5	0.4	53	0.3
Outside of summer (all) Summer (all)	60,481	1.6	15	0.1	97	0.0
	159,874	11.0	56	1.3	320	0.4

**Table 3:** Characteristics of patients seen in emergency departments (EDs) for sunburn, by gender, age, and distance from residence to consultation location, 2019 to 2022, for the 16 EDs selected in Occitanie.

	2019		2020		2021		2022		2019–2022	
	N	Part	N	Part	N	Part	N	Part	N	Part
**Gender**										
Women	75	55%	44	45%	52	50%	42	53%	213	51%
Men	61	45%	52	54%	53	50%	37	47%	203	49%
Unknown	0	0%	1	1%	0	0%	0	0%	1	0%
Sex ratio (M/W)	0.81		1.20		1.00		0.90		0.95	
**Age**										
0–9 years old	23	17%	19	20%	17	16%	20	25%	79	19%
10–19 years old	38	28%	30	31%	35	33%	21	27%	124	30%
20–39 years old	48	35%	30	31%	34	32%	26	33%	138	33%
40–59 years old	18	13%	11	11%	13	12%	7	9%	49	12%
60 years old and more	9	7%	6	6%	6	6%	5	6%	26	6%
Not available	0	0%	1	1%	0	0%	0	0%	1	0%
**Distance**										
0–19 km	72	53%	50	52%	41	39%	42	53%	205	49%
20–49 km	7	5%	8	8%	3	3%	7	9%	25	6%
50 km or more	47	35%	35	36%	57	54%	24	30%	163	39%
Outside the area	10	7%	4	4%	4	4%	6	8%	24	6%

## Data Availability

The data presented in this study are available on request from the corresponding author, but pharmacy data are not publicly available because of their link with the commercial activity of each pharmacy.

## Abbreviations

The following abbreviations are used in this manuscript:

ED	Emergency department
EVSs	Emergency visit summaries
ICD-10	International classification of diseases 10th revision
MA	Marketing authorization
NCOP	National Council of the Order of Pharmacists
OTC	Over-the-counter
PA_ED_	Proportion of suncare activity for emergency departments
PA_pharma_	Proportion of suncare activity for pharmacies
PA_SOSM_	Proportion of suncare activity for SOS Médecins procedures
PMS	Pharmacy management software
PPV	Positive predictive value
PRISME	Prevention and Impact of Sun Exposure on the Mediterranean Coast
SOSM	SOS Médecins associations
UV	Ultraviolet
WHO	World Health Organization
